# The total sale of prescription drugs with an abuse potential predicts the number of excessive users: a national prescription database study

**DOI:** 10.1186/s12889-015-1615-7

**Published:** 2015-03-25

**Authors:** Ingeborg Rossow, Jørgen G Bramness

**Affiliations:** Norwegian Institute for Alcohol and Drug Research (SIRUS), Øvre Slotts g 2b, P.O. Box 565, Sentrum, 0105 Oslo Norway; Norwegian Centre for Addiction Research, University of Oslo, Oslo, Norway; Center for Psychopharmacology, Diakonhjemmet Hospital, Oslo, Norway

**Keywords:** Prescription drugs, Pain relievers, Anxiolytics, Hypnotics, Abuse potential, Distribution, Excessive use, Drug sales

## Abstract

**Background:**

Prescription drug sales may vary considerably across regions and over time. This study aimed to assess whether there is an association between mean drug sales and prevalence of excessive use in a range of psychotropic prescription drugs with an abuse potential, and if so, whether the variation in mean drug sales mostly reflects variation in the prevalence of excessive use or mostly reflects variation in non-excessive use.

**Methods:**

Data on all filled prescriptions taken from the Norwegian prescription database for 10 drugs with an abuse potential (pain relievers, anxiolytics, and hypnotics) during one calendar year (2005) in Norway (n = 4 053 624) included number of defined daily doses (DDD). These were aggregated to individual level (n = 815 836) and county level (n = 19).

**Results:**

Analyses of individual level data showed that the distribution of drug use was skewed; those who used more than 365 DDD per year accounted for almost half of the sales of both anxiolytics and hypnotics. At the county level, the mean sales per inhabitant and the prevalence of excessive users were closely correlated, but both prevalence of non-excessive use and prevalence of excessive drug use were associated with the county-wise variation in mean drug sales.

**Conclusion:**

Despite a strong individual control of access to psychotropic drugs through health personnel’ prescribing, a small proportion of users account for a large fraction of the sales of these drugs. The sales vary significantly between regions and this variation is closely associated with the prevalence of excessive users. This suggests that sales figures as such may be used as an indicator to monitor variations in excessive use between regions and over time, and to evaluate interventions targeting over-prescription and excessive use.

## Background

Opioids, hypnotics, and anxiolytics are drugs mainly prescribed for the treatment of pain, insomnia or anxiety. In high income countries these drugs are widely used, however, they have a potential for abuse and are also used in excessive and non-therapeutic amounts by some patients [1-3]. Such excessive long-term use carries an elevated risk of many and diverse negative consequences. Beyond the risk of dependence and withdrawal symptoms that apply to all these drugs [4-6], the risks of long-term use of hypnotics and anxiolytics include also impaired cognitive functions and memory loss, impaired motor function and risk of falls and injuries, paradoxical effects such as anxiety, restlessness and insomnia, and aggravated dementia [7]. In most countries such drugs are therefore subject to formal control, most often in terms of a prescription system [8], a system of scheduling, and guidelines advocating restricted use of these drugs. Nevertheless, excessive use or abuse of pharmaceutical drugs also occurs under such regimes [9]. To some extent this can be attributed to diverted drugs and access from illegal markets [10], but some patients are also prescribed excessive amounts of these drugs [11]. It is the latter aspect of excessive drug use that will be addressed here.

The prescribing of opioids, hypnotics and anxiolytics varies enormously between countries, but a significant variation within countries is also observed, both between regions and over time [12]. For instance, prescriptions of benzodiazepines in Spain and the USA were four to five times higher than that in Germany [13] and in the USA, prescriptions of opioid analgesics varied by a factor of 12 between states [14]. Most often a prescription system will allow for generating wholesale statistics of drug sales at the aggregate level, but it would be important to know whether – or to what extent – the aggregate variation in these sales is indicative of variation in the prevalence of excessive use and abuse of these drugs.

As the prescription system is meant to prevent excessive use and thereby interfere with a ‘natural’ distribution of drug use, we may assume that the prescription system’s – and mainly the prescribers’ – ability to obtain this goal, will affect the distribution of drug use. Very different distribution patterns of use could therefore – in principle - underlie an overall variation in total sales. The overall variation could e.g. be due to variation in the number of users and the amount of use among non-excessive users with little or no variation in the prevalence of excessive use. Or vice versa; the overall variation in sales could be due to variation in the number of excessive users and their consumption. Or the variation in sales could be due to some combination of the two.

**Figure 1:**
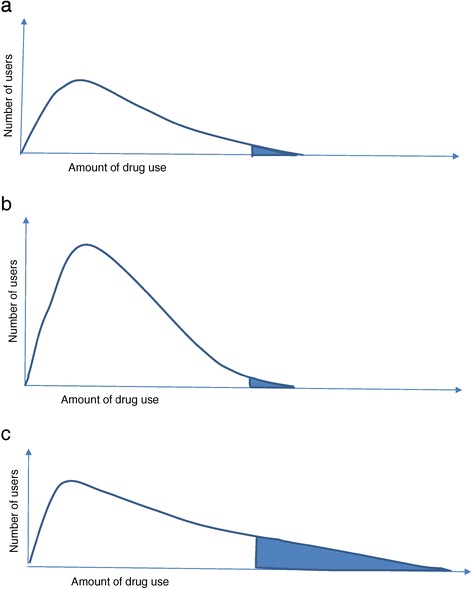
**Hypothetical distributions of drug use.** The area under the distribution curve is the total amount of drug use, which is larger in **b** and **c** than **a**. The filled area in the right tail of the distribution is the excessive drug use, which is the same in **a** and **b** and larger in **c**.

Obviously, the nature of this underlying distribution is not only of theoretical relevance, but also important from a public health perspective. Thus, if the variation could be ascribed mainly to the number of users, but not excessive use, high sales figures could suggest a higher morbidity rate in the studied population or a higher coverage of the target population. This is illustrated with the hypothetical drug use distributions in Figures 1a and b; the higher total drug use in 1b compared to 1a solely reflects an increase in number of non-excessive users, whereas the number of excessive users is the same. If, on the other hand, the variation in sales mainly reflected variation in excessive use, an increase in total drug use would reflect a more right-skewed distribution, as illustrated when comparing Figures 1a to c. In the latter scenario, this would mean that sales figures as such could be used to monitor variations in excessive use. If this were true, wholesale figures, more readily available, could be used to monitor differences in excessive use between regions and over time, and to evaluate interventions targeting over-prescription and excessive use.

So far, the distribution pattern of the consumption of prescription drugs with an abuse potential has scarcely been addressed in empirical studies. Indeed, surveys in general population samples and in institutionalized and other patient samples have demonstrated some aspects of the distribution of consumption, such as differences in prevalence of use and excessive use with respect to gender and age [15,16]. However, few studies have addressed a possible association between total sales of psychoactive drugs and prevalence of excessive use of these drugs, yet there is some indirect evidence suggesting this type of association. In the USA, both sales of opioids and treatment admissions for opioid abuse have increased significantly and in a parallel manner from 1999 to 2009 [17]. In a previous study [18], we examined the distribution pattern of one prescription drug with abuse potential (carisoprodol) and found that there was a strong and positive association between sales of carisoprodol and prevalence of excessive use. In other words, high total sales of this drug were indicative of high prevalence of excessive drug use, and vice versa. The aim of the present study was to pursue this finding and, by looking at the 10 most accessible prescription drugs with an abuse potential, to assess whether a close association between total drug sales and prevalence of excessive use could be found for a wider range of prescription drugs with an abuse potential, and if so, whether the variation in total drug sales mostly reflects variation in prevalence of excessive use or also reflects variation in non-excessive use.

## Methods

### Data source

Drug use data were retrieved from the Norwegian Prescription Database (NorPD). Access to the data was applied for and granted by the Norwegian Public Health Institute. The NorPD covers the entire Norwegian population (5.0 million inhabitants). From 1 January 2004, all pharmacies in Norway were obliged by law to submit electronic data every month on all prescriptions to the Norwegian Institute of Public Health. The NorPD contains information on all prescription drugs, reimbursed or not, dispensed at Norwegian pharmacies to individual patients who live outside institutions [19,20]. Over-the-counter drugs not requiring prescriptions are not registered. For each prescription the following data were collected and used in the present analyses: patients’ unique identifiers (encrypted), county of residence, and drug information; i.e. which drug based on the anatomical–therapeutic–chemical code (ATC-code) and the amount of the drug prescribed converted into number of defined daily doses (DDD). The DDD is the assumed average maintenance dose per day for the drug’s main indication in adults, it is assigned by the WHO collaborating centre and has been used in similar types of studies previously [21]. The data collected for the present study comprised 10 of the most widely used prescription drugs with an abuse potential in Norway [22], but not drugs on the narcotics list, like morphine and other opioids which are heavily restricted. The drugs were as follows (ACT-code, DDD and main use in parentheses): carisoprodol (M03BA02, 1400 mg, centrally acting muscle relaxant), codeine (N02AA59, 150 mg, opioid pain reliever), diazepam (N05BA01, 10 mg, anxiolytic), oxazepam (N05BA04, 50 mg, anxiolytic), nitrazepam (N05CD02, 5 mg, hypnotic), flunitrazepam (N05CD03, 1 mg, hypnotic), zopiclone (N05CF01, 7,5 mg, hypnotic), and zolpidem (N05CF02, 10 mg, hypnotic), that were filled in Norwegian pharmacies during the calendar year 2005. The year 2005 was chosen as this was the last year before carisoprodol came under scrutiny in the Norwegian market. The number of prescriptions for alprazolam (N05BA12, anxiolytic) and clomethiazole (N05CM02, hypnotic) were too few to analyse separately, but data on prescriptions for these drugs were included in summary measures of anxiolytic drugs and hypnotic drugs, respectively. The data set comprised a total of 4,053,624 prescriptions. Data on population figures were obtained from Statistics Norway.

### Excessive use of addictive drugs

Different definitions of excessive use of prescription drugs in general have been applied previously; for instance: a set amount of prescribed drug within a certain time period (e.g. more than 2 DDDs/day over a 30-day period) [23]; large prescriptions (e.g. more than twice the recommended amount in any prescription) [24]; and frequent prescribing (e.g. only a few days between prescriptions) [25]. In Norway, the guidelines for prescribing hypnotics and sedatives are that these drugs should be used only temporarily; daily use should be limited to the shortest possible time and no more than two to four weeks. Intermittent treatment is recommended for patients with chronic insomnia [26]. Carisoprodol was not to be used for more than 5–10 days. The use of opioids, including codeine use should be restricted to a minimum, but no strict time is indicated. Thus, we considered drug consumption exceeding daily use of a defined dose for a day (DDD) over an entire year as excessive use and we applied three indicators of excessive drug use for each drug; those who filled prescriptions during a year which amounted to: a) more than 365 DDDs, b) more than 730 DDDs and c) more than 1095 DDDs.

### Strategy of analyses

**Figure 2:**
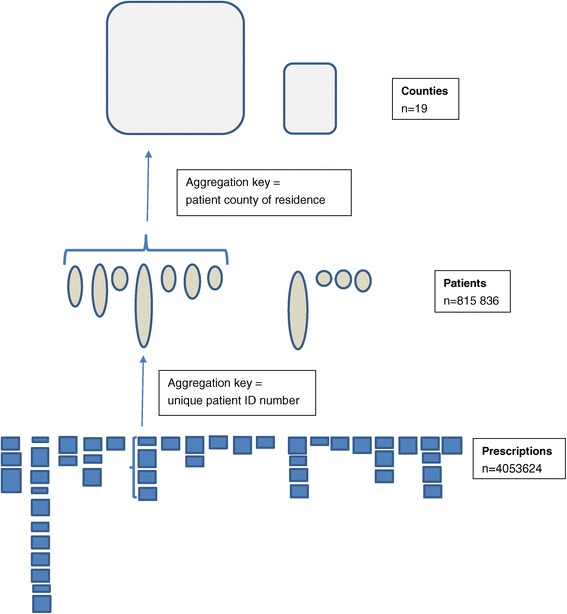
**Illustration of aggregation of filled prescriptions.** Each element illustrates the amount of a specific drug (in number of DDDs). The numbers are aggregated from prescriptions to patients and from patients to counties.

First, all prescriptions were aggregated to the individual level (n = 815 836), summarizing the amount of prescribed drugs in DDDs during the calendar year for each drug and drug group (Figure 2). By applying individual level data for the country as a whole, we described for each drug first the prevalence of past year users, the mean amount of drug use per inhabitant and year in the overall population and the mean and median amount per year among the users. Then, for each drug we described the prevalence of excessive users (applying various thresholds for excessive use) and the fraction of drug sales that these users accounted for.

Next, the individual level data were aggregated to the county level (n = 19) (Figure 2). We assessed whether there was any significant variation in mean drug sales and prevalence of excessive users between counties, applying maximum-minimum value ratios. We then assessed whether drug sales and prevalence of excessive users correlated, applying Spearman’s rank correlation coefficient. Due to low numbers, the third indicator of excessive drug use (>1095 DDDs/year) could not be included in the analyses at the county level.

Finally, we assessed to what extent the variation in drug sales between counties could be attributed to the prevalence of excessive users and the prevalence of non-excessive users. Linear ordinary least square (OLS) regression models were estimated, and input variables included by model fit criteria. In these analyses input variables comprised prevalence figures for three mutually exclusive categories of drug users; non-excessive users (<365 DDDs/year), moderately excessive users (365–730 DDDs/year), and highly excessive users (>730 DDDs/year). Beta coefficients were obtained to assess the relative importance of the input variables to predict variation in drug sales and model R square was obtained to assess the overall predictive value of the input variables included in each model.

All analyses were first carried out for each drug. Then all analyses were carried out for two main groups of drugs; all anxiolytic drugs (diazepam, oxazepam, and alprazolam) and all hypnotic drugs (nitrazepam, flunitrazepam, zopiclone, zolpidem, and clomethiazole), and finally all analyses were carried out for all 10 drugs together (carisoprodol, codeine, diazepam, oxazepam, alprazolam, nitrazepam, flunitrazepam, zopiclone, zolpidem, and clomethiazole).

## Results

### Distributions at the individual level

The prevalence of users and the mean sales per inhabitant varied significantly between the eight drugs; the highest prevalence of users was found for codeine (8.8% of the population) and zopiclone (6.3%) and the lowest for flunitrazepam (0.3%). Within one year, altogether 17.7% of the Norwegian population had filled one or several prescriptions for one or several drugs with an abuse potential. Mean sales per inhabitant per year were highest for zopiclone (10 DDD/inhabitant/year) and about equally low for carisoprodol, flunitrazepam and zolpidem (about 1 DDD/inhabitant/year) (Table 1). Mean consumption per user and year was particularly high for flunitrazepam and nitrazepam. For all drugs the mean consumption among the users exceeded the median considerably, implying a distribution heavily skewed to the right (Table 1).

**Table 1:** **Number of users (in 1000), prevalence of users per 1000 population, mean consumption per inhabitant and year in DDD, and mean and median consumption per user per year in DDD, by psychoactive drug category**

	**In total population**			**Among users**	
	**Number of users in 1000**	**Prevalence of users per 1000 population**	**Mean DDD per inhabitant per year**	**Mean DDD per year and user**	**Median DDD per year and user**
Carisoprodol	84	18.2	0.82	44.9	7.5
Codeine	406	88.2	4.35	49.3	12.5
Diazepam	153	33.2	3.74	112.9	45.0
Oxazepam	125	27.1	2.23	82.1	29.4
Nitrazepam	44	9.6	2.06	213.6	120.0
Flunitrazepam	14	3.0	0.80	263.9	180.0
Zopiclone	288	62.5	9.98	159.5	90.0
Zolpidem	42	9.1	1.11	120.8	45.0
Anxiolytics (including alprazolam)	266	57.8	6.8	118.4	37.5
Hypnotics (including clomethiazole)	361	78.4	14.0	177.8	100.0
All studied drugs	816	177.2	26.0	146.5	33.3

In line with the skew distributions we observed that a substantial proportion of users filled prescriptions exceeding 365 DDD per year, this proportion was particularly high for hypnotics (Table 2). The proportion of users filling prescriptions exceeding 730 DDD per year was not very high, but far from negligible, i.e. it was mostly around 1%, yet among flunitrazepam users, 6% filled prescriptions exceeding 730 DDD per year (Table 2). These excessive users accounted for a disproportionately high fraction of the total drug sales. Those who filled prescriptions exceeding 365 DDDs accounted for almost half of the total sales of anxiolytics and hypnotics, and those who filled prescriptions exceeding 730 DDDs accounted for almost one fourth of all sales of anxiolytics and for 13% of the total sales of hypnotics (Table 2). Even the very few who filled prescriptions exceeding 1095 DDDs accounted for a non-negligible fraction of the total drug sales; more specifically, they accounted for 15.8% of all anxiolytics sales and 6.4% of all hypnotics sales (Table 2).

**Table 2:** **Proportion of users with excessive use and the fraction of total consumption accounted for by excessive users by category of excessive use; > 365 DDD/year, > 730 DDD/year, > 1095 DDD/year, and by psychoactive drug category**

	**> 365 DDD**		**> 730 DDD**		**> 1095 DDD**	
	**Proportion of users (%)**	**Proportion of consumption (%)**	**Proportion of users (%)**	**Proportion of consumption (%)**	**Proportion of users (%)**	**Proportion of consumption (%)**
Carisoprodol	1.9	26.4	0.4	9.8	0.1	5.4
Codeine	2.5	29.9	0.5	10.2	0.1	4.5
Diazepam	7.6	43.3	1.6	15.4	0.5	7.2
Oxazepam	4.2	31.3	0.8	10.6	0.2	4.5
Nitrazepam	19.9	53.9	3.3	16.7	1.1	8.1
Flunitrazepam	24.9	62.0	6.1	27.9	2.2	14.9
Zopiclone	12.6	42.0	1.4	9.7	0.4	4.3
Zolpidem	8.5	41.3	1.3	12.6	0.4	6.5
Anxiolytics (including alprazolam)	7.4	47.4	2.1	23.9	0.9	15.8
Hypnotics (including clomethiazole)	15.1	47.6	2.1	13.2	0.7	6.5
All studied drugs	11.6	58.7	3.4	30.8	1.6	19.6

### Distributions and associations at the county level

The mean sales – and assumingly consumption – per inhabitant varied considerably across counties; the maximum-minimum ratio was of the magnitude of 2 to 3 for the various drug categories, and the variation in the prevalence of excessive users tended to be higher (Table 3). For all drug categories the mean sales per inhabitant was closely associated with the prevalence of users receiving more than 365 DDDs/year (Spearman’s rank correlation coefficients ranging between 0.87 and 0.99) (Table 3). There was also a positive and statistically significant correlation between mean sales and prevalence of users receiving more than 730 DDDs/year, yet of a somewhat more moderate magnitude (ranging between 0.52 and 0.89) (Table 3).

**Table 3:** **Variation in mean consumption and prevalence of excessive users (range and max-min ratio) between counties and correlations between mean consumption and prevalence of excessive users (>365 DDD/year; > 730 DDD/year) at county level**

	**Mean consumption per inhabitant**	**Prevalence w/ >365 DDD per 1000 population**	**Prevalence w/ >730 DDD per 1000 population**	**Correlations with mean consumption**	
	**range and max-min ratio**	**range and max-min ratio**	**range and max-min ratio**	**> 365 DDD Per 1000 inhabitants**	**> 730 DDD Per 1000 inhabitants**
Carisoprodol	0.40 - 1.09	0.14-0.48	0.02-0.15	0.87**	0.52*
	2.7	3.4	7.5		
Codeine	2.92 - 6.76	1.26-3.56	0.25-0.60	0.97**	0.72**
	2.3	2.8	2.4		
Diazepam	2.05-5.99	1.20-3.91	0.20-0.81	0.95**	0.82**
	2.9	3.3	4.0		
Oxazepam	1.11-3.83	0.45-2.04	0.10-0.38	0.96**	0.85**
	3.5	4.5	3.8		
Nitrazepam	1.11-3.49	1.00-3.30	0.11-0.48	0.99**	0.85**
	3.1	3.3	4.4		
Flunitrazepam	0.43-1.41	0.36-1.29	0.05-0.37	0.97**	0.85**
	3.3	3.6	7.4		
Zopiclone	6.07-13.17	3.70-11.11	0.37-1.42	0.99**	0.87**
	2.2	3.0	3.0		
Zolpidem	0.65-1.62	0.44-1.23	0.03-0.22	0.95**	0.88**
	2.5	2.8	7.3		
Anxiolytics (including alprazolam)	3.87-10.84	2.12-7.10	0.60-2.17	0.98**	0.89**
	2.8	3.3	3.6		
Hypnotics (including clomethiazole)	8.32-18.23	5.86-16.61	0.66-2.50	0.99**	0.86**
	2.2	2.8	3.8		
All studied drugs	16.25-33.86	13.28-29.04	2.98-9.65	0.99**	0.95**
	2.1	2.2	3.2		

Levels of statistical significance: *p < 0.05, **p < .01.

Finally, we addressed whether the variation in drug sales across counties was merely reflecting variation in excessive use or whether it also reflected the prevalence of users. For all drugs the prevalence of non-excessive users (<365 DDDs/year) and the prevalence of excessive users both contributed significantly to explain the variation between counties in drug sales per inhabitant. The beta coefficients – and thus the relative importance – of prevalence of moderately excessive users (365–730 DDDs/year) varied somewhat across drug categories and were higher than that for non-excessive users and highly excessive users (>730 DDDs/year) with respect to codeine, anxiolytics and hypnotics (Table 4). For five of the eight drugs (diazepam, nitrazepam, flunitrazepam, zopiclone and zolpidem) and for both drug groups (anxiolytics and hypnotics), even the prevalence of highly excessive users contributed significantly to the variation in drug sales between counties (Table 4).

**Table 4:** **Associations between mean sales of psychoactive drugs (in DDD/1000 inh/year) and prevalence of users and of excessive users by type of psychoactive drug**

	**Prevalence of non-excessive users**				**Prevalence of excessive users > 365 DDD, < 730 DDD**				**Prevalence of excessive users > 730 DDD**				**Model R** ^**2**^
	**Regr coeff**	**SE**	**p-value**	**Beta**	**Regr coeff**	**SE**	**p-value**	**Beta**	**Regr coeff**	**SE**	**p-value**	**Beta**	
Carisoprodol	0.026	0.007	.002	.543	1.243	0.412	.008	.439	-	-		-	0.864
Codeine	-	-		-	1.761	0.120	<.001	.961	-	-		-	0.923
Diazepam	0.066	0.006	<.001	.403	0.771	0.057	<.001	.483	1.176	0.172	<.001	.231	0.989
Oxazepam	0.052	0.004	<.001	.536	1.058	0.078	<.001	.490	-	-		-	0.995
Nitrazepam	0.105	0.010	<.001	.353	0.583	0.038	<.001	.507	1.168	0.109	<.001	.182	0.998
Flunitrazepam	0.142	0.013	<.001	.351	0.427	0.048	<.001	.331	1.470	0.076	<.001	.464	0.993
Zopiclone	0.070	0.015	<.001	.192	0.851	0.055	<.001	.749	0.602	0.251	.029	.085	0.993
Zolpidem	0.069	0.007	<.001	.409	0.693	0.063	<.001	.447	1.305	0.229	<.001	.242	0.988
Anxiolytics (also including alprazolam)	0.024	0.006	.002	.200	1.021	0.148	<.001	.526	1.489	0.246	<.001	.353	0.979
Hypnotics (also including clomethiazole)	0.072	0.019	.002	.182	0.747	0.053	<.001	.676	1.042	0.182	<.001	.182	0.994
All studied drugs	0.051	0.018	.012	.118	0.707	0.087	<.001	.449	1.437	0.138	<.001	.485	0.990

Unstandardized regression coefficients, standard errors of estimates, standardized regression coefficients (beta) and model R square obtained in linear regression models.

Only statistically significant associations are presented.

## Discussion

This study has demonstrated that across a wide a range of prescription drugs with an abuse potential the distribution of consumption as measured by filled prescriptions was heavily skewed, and that the relatively few excessive users accounted for a disproportionately high fraction of the drug sales. This was true for drugs like benzodiazepine anxiolytics and hypnotics, z-hypnotics and weak opioid analgesics. Moreover, the sales and prevalence of excessive users varied significantly between counties and there was a strong positive correlation between the two. Thus, the prevalence of excessive users accounted for much of the variation in drug sales, yet the prevalence on non-excessive users also contributed to the variation in drug sales between counties.

Our observations of a very skewed distribution of use of prescription drugs with an abuse potential resemble those from some previous studies of such drugs [27]. Moreover, these findings add to a meagre literature on the distribution of prescription drug use and the association between drug sales on the one hand and indicators of excessive use on the other. It demonstrates that what we found earlier for carisoprodol alone [18] is applicable to a series of drugs with abuse potential, indicating that whole sales of various drugs with an abuse potential is closely related to excessive use. This is in line with previous observations of a correlation between wholesale of prescription opioids and treatment admissions [17], and relevant to the observed correlations between wholesale and overdoses of opioid pain relievers [17,28] and of carisoprodol [29,30].

### Study strengths and limitations

The major strength of this study is that we have a complete prescription register providing data on all psychotropic drug sales in the non-institutionalized population. The data are not subject to attrition bias and reporting bias as in population surveys. The data are accurate with respect to drug, drug category and dispensed amounts as opposed to self-reported data. In this study we have large numbers of patients who have filled prescriptions, which allows for comparisons of distribution measures across geographical units, i.e. counties.

The study limitations include not having a clinical diagnosis of drug abuse, but having to rely on figures of filled prescriptions. We have attempted to compensate for this by introducing a conservative operationalization of excessive use. This implies that among those categorized as ‘non-excessive users’, a fraction have filled prescriptions exceeding the Norwegian national guidelines limits for prescriptions of opioids, benzodiazepines and z-hypnotics. For this reason, the prevalence of excessive use of these drugs may have been underestimated. Another limitation is that filled prescriptions may not necessarily have been entirely consumed by the recipients. Some drugs may have been diverted (sold or given away to others) or been lost, wasted or returned unused to the pharmacy and this is probably more likely to have occurred among excessive users. However, drug users may have supplemented their drug access with illegal or diverted drugs. Thus, the prescription register base data do not provide a perfect and accurate picture of excessive use (whatever the definition is).

Finally, the data set dates 10 years back in time. While figures for total sales and prevalence of excessive use for each drug may change over time, for instance in response to change in scheduling of particular drugs, the mechanisms underlying the strong associations between total sales and excessive use are probably less subject to change over time. In fact, the 10 drugs we studied differed hugely with respect to total sales and prevalence of users and for all these we found strong associations between sales and excessive use. This suggests that the strength of these associations is not very sensitive to the level of sales. Yet, further studies are needed to assess the generalizability of the findings both over time and across jurisdictions.

### Implications

These findings are important as they show that changes – or differences – in excessive use of prescription drugs can be predicted with some accuracy on the basis of sales data only. Consequently, this may also apply to the prevalence of problems related to such use. In the absence of a programme for individual monitoring of prescription drug use, drug sales at the aggregate level thus seems to provide a useful proxy tool for assessment of variation in excessive drug use and may be a useful indicator of prevalence of excessive use in studies evaluating possible impacts of policy changes or other interventions targeting excessive psychotropic drug use. For instance, the increase in problems connected with prescription opioids in the USA [31] could have been predicted by the sharp increase in sales of these drugs alone [32].

Further research in this area is warranted and may take several directions. For instance, replications of the present study on prescription drugs with an abuse potential could address external validity of the present findings. It would be important to address whether these distributional patterns apply to other - often widely used - addictive prescription drugs, such as oxycodone and methylphenidate, and whether they apply also to non-addictive drugs. Moreover, it will be highly important to assess the prescribers’ role in excessive drug use. For instance, more knowledge is needed regarding the extent of ‘doctor shopping’ among excessive drug users, as well as a better understanding of the process and interaction between patient and prescriber that underlie the development of excessive use of prescribed drugs.

## Conclusions

Despite that access to psychotropic drugs is subject to a strong individual control through clinicians’ prescribing, a non-negligible proportion of psychotropic drug users have filled prescriptions within a year clearly indicating excessive use, and these excessive users account for a disproportionately large fraction of the sales of these drugs. The sales vary significantly between regions and this variation is closely associated with the prevalence of excessive users. This suggests that sales figures as such may be used as an indicator to monitor variations in excessive use between regions and over time, and to evaluate interventions targeting over-prescription and excessive use.

## Abbreviations

ATC: Anatomical Therapeutic Chemical (Classification System)
DDD: Defined Daily Doses
NorPD: Norwegian Prescription Database
